# Differences in Utilization of Medical and Dental Services among Homeless People in South Korea

**DOI:** 10.3390/ijerph17155304

**Published:** 2020-07-23

**Authors:** Seung-Hyun Lee, Jae-In Ryu, Se-Hwan Jung

**Affiliations:** 1 Department of Preventive and Public Health Dentistry, College of Dentistry, Gangneung-Wonju National University, Gangneung-si 25457, Korea; leesh.dmd@gmail.com (S.-H.L.); feeljsh@gwnu.ac.kr (S.-H.J.); 2 Department of Preventive and Social Dentistry, College of Dentistry, Kyung Hee University, Seoul 02447, Korea

**Keywords:** utilization of health service, medicaid program, outreach program, homeless

## Abstract

(1) Background: Homelessness contributes to both needs for care and barriers to access. This study aimed to explore the utilization of medical or dental services using Andersen’s model for a vulnerable population of homeless in South Korea. (2) Methods: The data were applied from the first national survey for homeless people in South Korea, 2016. Totally 2032 persons participated in the interview survey. This study team requested the raw data through the public portal and analyzed them. (3) Results: The participants who were homeless for more than ten years, staying in small rooming house or shelter, non-employed, earning less than 500,000 won per month, and having a medical condition showed a significantly higher chance of using Medicaid. The use of outreach programs had a significant relationship with gender, duration of homelessness, and monthly income. Among dental patients, the homeless who did not consume alcohol, stayed in a shelter, and were employed had higher chances of using dental service. (4) Conclusions: Medicaid service was strongly related to enabling factors but outreach programs with predisposing factors. Dental service showed strong relationships with the enabling domain, but the pattern was opposite: the jobless had less chance to avail it. The policymakers need to consider these domains of service utilization to provide equitable access to healthcare services.

## 1. Introduction

The United Nations (UN) Economic and Social Council announced that “affordable housing and social protection systems for all to address homelessness” should be the priority theme because it threatens family, communities, and human rights [1]. The U.S. Department of Housing and Urban Development (HUD) defines “homeless” as an individual or family who lacks a fixed, regular, and adequate nighttime residence. Even though it is hard to unify the international definition of homelessness, the European Federation of National Organizations Working with the Homeless (FEANTSA) developed a typology of homelessness. The European Typology of Homelessness and Housing Exclusion (ETHOS) classifies living situations of homelessness or housing exclusion as roofless, houseless, insecure housing, and inadequate housing [2]. Recently, they developed a shorter version, “ETHOS Light”, with the category of the homeless consisting of people living rough, in emergency accommodation, living in accommodation for the homeless, institutions, or non-conventional dwellings due to lack of housing [3]. The homeless on any one night are more than 400,000 in the European Union (EU) and 60,000 in the United States of America (USA) [4]. The homeless population was estimated at less than 1% of the population and the rates of people experiencing housing instability are much higher, 2–25% of the population [5].

The risks of health in homeless people are high as they are exposed to various dangerous environments. The age and gender standardized mortality ratios (SMR) of the homeless ranged from three to eleven compared to that of the other citizens [6,7,8]. Physical and psychiatric health problems exist throughout their life [9]. They are fragile for infectious diseases such as tuberculosis, hepatitis C, and HIV [10] and traumatic injuries needing emergency service [11]. Chronic diseases could stack up when they get old and make it difficult to manage the disease and pains [12]. Oral health is poor [13], which is one of the chronic diseases, having more decayed teeth [14], tooth loss, and oral pain [15,16]. It leads to poor quality of life—related to oral health with the psychological disability, social disability, and handicap [17,18]. Homelessness contributes to both the need for care and barriers to access it [19,20]. The health status of the homeless and their access to care service are much below those of the general population [21,22]. Emergency services are common to relieve pain [23].

Recently, there was a discussion about delivering oral healthcare service for socially excluded people who are in danger of “extreme oral health” [24]. “Social exclusion” separates them from mainstream society, stigmatizes with othering, and cuts them off from social systems [6,25]. “Inclusion oral health” begins from an understanding of how social exclusion is produced and experienced, and how forms of exclusion and discrimination intersect to compound oral health outcomes. It focuses on developing innovative inter-sectoral solutions to tackle the inequalities of people enduring extreme oral health. There are several ways of turning exclusion into inclusion, such as Medicaid or outreach programs, which have been implemented in many countries. Homeless people who registered in Medi-Cal received oral healthcare services more frequently [26]. Nevertheless, homeless people still experienced difficulties in accessibility [27]. Most of them faced financial barriers and felt unwelcome when they visit a dental clinic [28].

Several studies have adopted Andersen’s framework to find critical domains in healthcare utilization by the vulnerable populations [29,30]. Andersen’s behavioral model categorized them as predisposing characteristics, enabling resources, and need domains to access medical care [31]. The author argued that these domains are a series of causal relationships, that predisposing factors might be exogenous, enabling resources are essential but insufficient, and need can be defined in an actual situation. Gelberg’s team applied this model to homeless people to find the predictors of health service use and physical health outcomes [29]. They concluded that enabling factors, such as having a regular source of care, made health outcomes better. Even in the study by Varga and colleagues, the enabling and need domains showed a significantly higher prediction for the utilization of health service after adjustments [30]. These behavioral models aimed to find a way to improve the inequalities of utilization. Many researchers have suggested that it is necessary to provide a regular source of care within the community to enhance fairness [28,29]. If people can use the care service when they need it, their health could be improved and it might alleviate the inequalities of the marginalized populations to enhance equity.

In South Korea, the number of homeless people increased after the economic crisis in the 1990s. Most of them experienced financial and nonfinancial barriers in accessing health services, although they had severe health problems [32]. If they or their families have a certain amount of income, they can have National Health Insurance (NHI), which covers most of the country’s population, and whose major source of financing is contributions from the insured [33]. The homeless people might access outreach programs run by voluntary organizations offering medical or dental services with no charge or restriction. Homeless Medicaid was established in 2012 to support the homeless who could not use the general Medicaid service due to, for example, expunged identification. Medicaid program is public assistance operated from the tax to guarantee the minimum living conditions. All the Medicaid services from the government are provided only to qualified recipients. However, the service still has several problems such as high hurdles presented by selection criteria, limited coverage of the service, and few service providers even in public institutions [34].

This study aimed to explore the utilization of medical or dental services using Andersen’s model for a vulnerable population of homeless in South Korea to find a solution to their difficulties in accessing these services.

## 2. Materials and Methods

### 2.1. Study Participants

The first national survey for homeless people in South Korea performed a two-stage sampling in 2016 [35]. First, the size of the homeless population was estimated with Point-In-Time (PIT) counting methods by institutions or small rooming houses for the sheltered, and the unsheltered on the street. This kind of method needs to be objective, consistent, and periodical. The date and time of the survey were selected by weather, the pattern of moving, and the active support of investigators in the case of the street team. The first survey to count the homeless people was conducted on October 20 from 0 a.m. to 5 a.m., when it is not too hot or cold and people gather as usual. Additionally, it was before they received beneficiaries’ benefits because, after that, they moved to other places, such as motels. The conference or meeting schedules of involved institutions were considered carefully to facilitate their participation. The investigators were trained for two days before the survey. Each survey team consisted of an on-site expert and an investigator from a research institution. The key institutions collected information about the region from local organizations. In all, 138 institutions participated: 19 shelters, 62 self-sufficiency institutions, 35 rehabilitation facilities, and 22 healthcare facilities. Ten counseling centers for the residents of the small rooming houses were selected for the investigators to use as bases for the survey. The survey team visited 1254 key points on the street where the homeless people were found previously through programs such as outreach support. They conducted pilot studies in these regions to minimize unexpected risks before the survey. In the second stage, the samples were selected by the stratification of each of the five regions in terms of the population in the street, institution, or small rooming house. The investigators were trained for interviews two days before the survey. They were on-site experts or investigators from research institutions. They conducted in-depth interviews with prepared questionnaires on living conditions, psychological status, and the need for healthcare services. The participants were given a brief introduction of the study, and their consent sought for the interview. Only those who agreed to participate were included in the study. The participants received rewards of about five dollars and portable hot packs. Finally, 2032 persons participated in the interview survey of the homeless people in South Korea. Most national survey data are open to researchers in South Korea. This study team requested the raw data through the Health and Welfare Data Portal [36] and analyzed it for this study. The Institutional Review Board at Gangneung-Wonju National University Dental Hospital reviewed and approved this secondary data analysis (GWNUDH-IRB2019-A015). 

### 2.2. Study Variables and Measurement

The sociodemographic variables included the categories of the Gelberg–Andersen behavioral model for health service utilization in vulnerable populations [37]. The predisposing domain included gender, age, education, duration of the homeless period, and health-related behaviors, such as drinking and smoking. The enabling domain included housing, employment, and monthly income. The need domain included perceived health status, medical, and dental diseases. General medical diseases included, metabolic, heart, otolaryngological diseases, and so on, referring to the categories from Korea National Health and Nutrition Examination Survey (KNHANES) [38] and Korean Community Health Survey (CHS) [39]. The most frequent diseases that homeless people experienced were metabolic disease, mental disorder, and dental diseases. The outcome variables were the utilization of medical services, such as Medicaid or outreach programs, and dental service. Medicaid could be general Medicaid or Homeless Medicaid. Only those who answered they had a dental disease were asked about the utilization of any kind of service.

### 2.3. Statistical Analysis

The utilization of medical services such as Medicaid or outreach programs and dental services were examined by chi-square tests according to the indicators of three domains of the Gelberg–Andersen behavioral model for health service utilization. To identify the independent effects of each domain to medical and dental services, Poisson regression models were estimated [40]. Each domain was included in the analysis step-by-step to evaluate the effect on the dependent variable.

- Unadjusted Model
- Model 1: adjusted with predisposing domain
- Model 2: adjusted with predisposing and enabling domains
- Model 3: adjusted with predisposing, enabling, and need domains

This modeling approach allows the comparison of predisposing, enabling, and need variables to distinguish which domains better predict the utilization of the services. The variables that were significant at p-value less than 0.05 were determined to have significant effects on the dependent variable of utilization. Data analysis was carried out using the STATA version 15.1 statistical software package (StataCorp, College Station, TX, USA).

## 3. Results

The general characteristics of the study participants are shown in Table 1. The percentage of men was four times that of women. Most homeless people were in their fifties or sixties and stayed in shelters. Half of the participants had been homeless for less than a decade. More people indulged in smoking than drinking, both behaviors hazardous to health. The unemployed constituted 60.7% and 68.2% of them earned less than 500,000 won a month. More than three-quarters of the homeless people had at least one disease. All the indicators of predisposing, enabling, and need domains were significantly related to the utilization of Medicaid service, except subjective health. The duration of homelessness, housing status, and having a disease showed a statistically significant relationship with outreach programs. The existence of dental diseases had a strong relationship with the usage of Medicaid services but not with outreach programs.

A quarter of the participants reported that they experienced some dental disease in the previous year (Table 2). Only the age, the subjective health status, and the existence of medical disease were factors significantly related to dental disease. Older persons, and those who perceived they had bad health conditions or had any kind of diseases showed higher tendencies to be diagnosed as having dental diseases. Of the people who had dental disease, 61.3% visited dental clinics after diagnosis. The homeless who did not drink stayed in a shelter and were employed had more tendencies to go to the clinic for dental treatments. 

Table 3 presents the correlations between the demographic and single-item variables. The duration of homelessness in the predisposing domain had strong relationships with all the variables in this study except dental disease and dental service. Enabling factors of service utilization showed significant tendencies to correlate with predisposing domains of this study. For example, employment and income levels were strongly related to all the predisposing domains. Among the need domain, medical conditions were closely associated with all the other factors in this model except income and dental service. Medicaid showed similar patterns of the relationship as medical diseases for the variables.

Table 4 shows the prevalence ratios (PRs) of the utilization of medical welfare services and dental services by domains of the Gelberg–Andersen behavioral model. In Medicaid, the participants who were homeless for more than ten years, staying in small rooming house or shelter, non-employed, earning less than 500,000 won per month, and having a medical condition showed a significantly higher chance of using the service even after full adjustment. Housing status made the biggest and most significant gaps: 2.19 times more chance for small rooming house residents and 2.45 times for shelter occupants compared to the street people. Education, the habit of consuming alcohol or smoking, and the existence of dental diseases showed statistical significance in the unadjusted models, but they disappeared after full adjustments. The use of outreach programs had significant relationships with gender, duration of homelessness, and monthly income. The men, being homeless more than ten years, or earning over 500,000 won, showed a higher chance of using outreach services. Housing, especially staying in a shelter, and the existence of diseases showed statistical significances, but they disappeared in Model 3. However, significant differences appeared from Model 2, which included enabling domains. Among dental patients, the homeless who did not consume alcohol, stayed in a shelter, and were employed had higher chances of using dental service. Housing status especially made the biggest and most significant gaps in using dental services, with the homeless staying in a shelter being 1.55 times more likely to use them than the street people. Employment was strongly related to the utilization of dental service as well as Medicaid. However, the pattern of prevalence was the opposite, as the jobless made more use of Medicaid services but had less opportunity for dental services. The pseudo R^2^ value was the biggest within Model 3, which adjusted full domains, 0.0151.

## 4. Discussion

There were differences in the utilization of medical welfare services and dental services among homeless people in South Korea. According to the Gelberg–Andersen behavioral model for vulnerable populations, three domains of predisposing, enabling, and need were analyzed for each service. Medicaid showed a significant relationship with enabling factors and outreach programs with predisposing factors. Contrary to Medicaid, dental service was significantly related to enabling domains.

First, Medicaid service was strongly related to enabling factors, such as housing status, employment, and income level. This result was similar to the findings of other studies that the enabling factors were essential to increase the availability of dental service [41]. Especially housing status was associated with the utilization of the service; for example, the people in the shelter had more than double the chances of using Medicaid service than those in the street. This might be closely associate with the regulation for homeless people in South Korea. The qualification for Homeless Medicaid was designated only for the residents staying at shelter or self-support facilities at least three months before the application [42]. Housing is the main issue related to homelessness [2] and their health [43,44,45]. According to Maslow’s hierarchy of needs, physical and safety needs are the most basic needs [46,47]. The “rooflessness” is the condition of the people without a shelter of any kind and sleeping rough. These people had less chance to use Medicaid. Sometimes they were not aware of their eligibility or the benefit of the public services [48,49]. Most studies pointed out that primary healthcare or regular service is an important factor for the homeless to eliminate imminent danger to health [50,51,52]. They recommended Medicaid or outreach services be expanded [53] to support housing or to help saving medical expenses [54]. In addition, in the final adjusted model, those homeless for more than ten years, not employed, earning less than 500,000 won, or having a medical condition were more likely to use Medicaid. The minimum earning of 500,000 won is similar to the federal poverty level (FPL) for Medicaid in the U.S [55]. Thus, Medicaid services could be used for people who need it more. 

Secondly, predisposing factors had a significant association with the utilization of outreach programs. Women and the minimum income earners had less chance to use outreach services while those homeless for more than ten years had 1.22 times more chance. This is the opposite findings of previous studies in which, women showed higher tendencies to use healthcare services [56]. Lewis pointed out that there was a significant unmet need for medical care of homeless women [51]. It was argued that the unmet need was higher when women thought there were barriers in access to care. Tam and colleagues suggested that women with mental illness reported a higher rate of using medical services [57]. Thus, the less utilization of the outreach services might come from the barriers in being treated. For example, women with illness lacked information or were not recommended to visit the services which are mostly managed by volunteers. Those who had been homeless for a longer duration and had higher income had more tendencies to visit outreach, indicating that this service might be run on arbitrary criteria rather than a clear standard for inclusion such as Medicaid. The volunteer works are essential but need a detailed framework to deliver to the needed.

Thirdly, as in Andersen’s assumption, dental service showed strong relationships with the enabling domain [31]. The people staying in shelters showed 1.55 times higher prevalence ratios compared to the street people. However, the homeless who were unemployed showed less tendency to use dental service, but not so far Medicaid. A majority of researchers suggested that there were lots of barriers to dental care such as fear, lack of money, not prioritizing dental problems, or chaotic health conditions which make patients hard-to-reach [22,28,58,59,60,61,62] even though they are in considerable need of care [63,64,65,66,67,68]. The homeless people’s unmet need for dental service—their inability to use a dental service despite having a dental disease—was 38.7%. This was higher than the unmet need for dental services (26.0%) and the unmet need for medical service (8.8%) in the national survey [38]. To reduce the gap between the need and service utilization, the targeted services in the fixed places continued their visits [69,70,71], and the integration and collaboration with other areas of services provided to the homeless were suggested [61,72]. Alcoholic drinking from the predisposing domain also showed a negative relationship with the utilization of dental service. In general, homeless staying in the street showed a higher tendency to be a heavy drinker [73]. The housing status was negatively associated with drinking habits as in other studies (Table 3), which means that the street people with a higher tendency to drink had less chance to receive dental services. 

This study has several limitations. Above all, medical diseases of the need domain had a significant association only with Medicaid. Homeless people usually had multiple complex chronic diseases and a high level of healthcare needs [74]. Nonetheless, medical conditions did not show a significant relationship with outreach programs or dental services. It could be interpreted that these services were not related to their medical conditions. A possible reason for this would be that the need domain did not have as strong a relationship as predisposing or enabling factors. Conversely, medical conditions were too common to be a specific domain of utilization. In this study, over three-quarters of the homeless people had any disease. In addition, it might come from the inaccuracy of the subjective questionnaire answers, which substituted medical examination. The results show that the shelter dwellers had a higher number of diseases. Generally, the homeless in the street showed a higher tendency to have medical conditions, including mental disorders, than those in the shelter [75,76]. They might have more diseases than they said, due to a lack of chance to be diagnosed. In the next survey, medical examinations need to be conducted together with subjective questionnaires. The other limitation was that only those with diagnosed dental diseases were asked about the utilization of dental services. This restricted the questionnaire to the unmet need for dental treatment. It does not include a dental visit for any other reason. In addition, the survey focused merely on the status of utilization, ignoring how difficult it is to receive the service and why. Even though it was the first national survey about homeless people in South Korea, it needs to gauge the magnitude of their problems of access and the way to find the solutions. These could make the service more accessible to fragile people. Lastly, the environmental factors in the dynamic model of health services were not included in this study. Even though the range of the service was limited to the public sector, in recent years, it is necessary to cover comprehensive environmental domains such as healthcare systems. The questionnaires need to extend these kinds of mechanisms for the service in future studies.

## 5. Conclusions

The utilization of medical welfare services for homeless people showed different relationships. Medicaid was significantly associated with enabling domains of housing status, employment, and income; the poorer received more chances to use this service. Housing showed the most significant relationship with service utilization such as Maslow’s hierarchy of needs with the physical and safety needs as basic needs. Outreach programs showed a strong relationship with the predisposing factors such as gender and duration of homelessness. Dental service had a significant relationship with the enabling domains as Medicaid, but the pattern was opposite, and the jobless had less chance to avail it. The policymakers need to consider these domains of service utilization to provide equitable access to healthcare services.

## Figures and Tables

**Table 1:** The utilization of medical welfare service by three domains of Gelberg–Andersen behavioral model in homeless people, S. Korea.

Variables	Total *N* = 2032		Medicaid *N* = 2020		Outreach Programs *N* = 2026	
	*n*	(%)	*n*	(%)	*n*	(%)
Total	2032	(100.0)	1439	(71.2)	1437	(70.9)
**Predisposing**						
Sex			*p < 0.001*		*p = 0.943*	
Men	1634	(80.4)	1111	(68.3)	1156	(71.0)
Women	398	(19.6)	328	(83.3)	281	(70.8)
Age			*p < 0.001*		*p = 0.793*	
<50	545	(26.9)	338	(62.7)	388	(71.5)
≥50	1483	(73.1)	1100	(74.5)	1048	(71.9)
*missing*	*4*		*1*		*1*	
Education			*p < 0.001*		*p = 0.313*	
≥High School	868	(43.3)	581	(67.2)	605	(69.9)
≤Middle School	1139	(56.8)	849	(75.1)	818	(72.0)
*missing*	*25*		*9*		*14*	
Duration (years)			*p < 0.001*		*p < 0.001*	
<10	934	(50.1)	607	(65.4)	602	(64.7)
≥10	929	(49.9)	733	(79.3)	735	(79.3)
*missing*	*169*		*99*		*100*	
Drinking			*p < 0.001*		*p = 0.059*	
No	1214	(59.8)	940	(77.9)	877	(72.5)
Yes	817	(40.2)	499	(61.5)	559	(68.6)
*missing*	*1*				*1*	
Smoking			*p < 0.001*		*p = 0.231*	
No	786	(38.7)	613	(78.6)	568	(72.5)
Yes	1246	(61.3)	826	(66.6)	869	(70.0)
**Enabling**						
Housing Status			*p < 0.001*		*p < 0.001*	
Street	299	(14.7)	73	(24.5)	188	(63.1)
Small Room	302	(14.9)	193	(64.1)	200	(66.2)
Shelter	1431	(70.4)	1173	(82.5)	1049	(73.6)
Employed			*p < 0.001*		*p = 0.122*	
Yes	795	(39.3)	525	(66.3)	547	(69.0)
No	1226	(60.7)	909	(74.7)	882	(72.2)
*missing*	*11*		*5*			
Income (1000 won)			*p < 0.001*		*p = 0.472*	
≥500	525	(31.8)	316	(60.5)	382	(72.9)
<500	1124	(68.2)	893	(80.0)	798	(71.2)
*missing*	*383*		*230*		*8*	
**Need**						
Subjective Health			*p = 0.367*		*p = 0.222*	
Good	724	(35.7)	503	(70.0)	524	(72.6)
Bad	1304	(64.3)	932	(71.9)	910	(70.0)
*missing*	*4*		*4*		*3*	
Medical Diseases			*p < 0.001*		*p < 0.001*	
No	467	(23.0)	250	(53.9)	299	(64.2)
Yes	1565	(77.0)	1189	(76.4)	1138	(73.0)
Dental Diseases			*p = 0.019*		*p = 0.438*	
No	1459	(71.9)	1011	(69.7)	1038	(71.4)
Yes	571	(28.1)	426	(75.0)	397	(69.7)
*missing*	*2*		*2*		*2*	

**Table 2:** The dental diseases and the utilization of dental services by three domains of Gelberg–Andersen behavioral model in homeless people, S. Korea.

Variables	Dental Diseases *N* = 2030		Dental Devices *N* = 569	
	*n*	(%)	*n*	(%)
Total	571	(28.1)	349	(61.3)
**Predisposing**				
Sex	*p = 0.704*		*p = 0.110*	
Men	456	(27.9)	271	(59.7)
Women	115	(28.9)	78	(67.8)
Age	*p = 0.001*		*p = 0.877*	
<50	122	(22.4)	75	(62.0)
≥50	447	(30.2)	273	(61.2)
*missing*	*2*		*1*	
Education	*p = 0.704*		*p = 0.780*	
≥High School	250	(28.9)	152	(60.8)
≤Middle School	320	(28.1)	197	(62.0)
*missing*	*1*			
Duration (years)	*p = 0.877*		*p = 0.922*	
<10	267	(28.6)	164	(61.7)
≥10	262	(28.3)	162	(62.1)
*missing*	*42*		*23*	
Drinking	*p = 0.570*		*p = 0.001*	
No	347	(28.6)	231	(66.8)
Yes	224	(27.5)	118	(52.9)
Smoking	*p = 0.490*		*p = 0.169*	
No	214	(27.3)	139	(65.0)
Yes	357	(28.7)	210	(59.2)
**Enabling**				
Housing Status	*p = 0.081*		*p < 0.001*	
Street	68	(22.7)	24	(35.3)
Small Rooming	88	(29.1)	44	(50.0)
Shelter	415	(29.0)	281	(68.0)
Employed	*p = 0.219*		*p = 0.044*	
Yes	235	(29.6)	154	(66.1)
No	331	(27.0)	191	(57.7)
*missing*	*6*		*4*	
Income (1000 won)	*p = 0.201*		*p = 0.330*	
≥500	164	(31.2)	95	(58.3)
<500	316	(28.2)	198	(62.9)
**Need**	91		*56*	
Subjective Health	*p = 0.001*		*p = 0.198*	
Good	171	(23.6)	111	(65.3)
Bad	399	(30.7)	237	(59.6)
*missing*	*1*		*1*	
Medical Diseases	*p < 0.001*		*p = 0.968*	
No	78	(16.7)	48	(61.5)
Yes	493	(31.5)	301	(61.3)

**Table 3:** Correlations among variables in homeless people, Seoul. Korea

	1	2	3	4	5	6	7	8	9	10	11	12	13	**14**	**15**
**Predisposing**															
1. Sex	-														
2. Age	0.00	-													
3. Education	0.11 ***	0.21 ***	-												
4. Duration	0.14 ***	0.10 ***	0.17 ***	-											
5. Drinking	−0.25 ***	−0.05 *	−0.08 ***	−0.09 ***	-										
6. Smoking	−0.43 ***	0.02	−0.07 **	−0.11 ***	0.28 ***	-									
**Enabling**															
7. Housing Status	0.16 ***	0.03	0.02	0.08 ***	−0.25 ***	−0.10 ***	-								
8. Employed	0.08 ***	0.16 ***	0.13 ***	0.16 ***	−0.16 ***	−0.11 ***	−0.06*	-							
9. Income	0.16 ***	0.06 **	0.08 **	0.16 ***	−0.12 ***	−0.11 ***	0.35 ***	0.09 ***	-						
**Need**															
10. Subjective Health	−0.04	0.10 ***	0.02	0.05 *	−0.02	−0.03	−0.11 ***	0.08 ***	−0.12 ***	-					
11. Medical Disease	0.08 ***	0.13 ***	0.05 *	0.14 ***	−0.13 ***	−0.07 **	0.22 ***	0.06 **	0.04	0.23 ***	-				
12. Dental Disease	0.01	0.08 ***	−0.01	−0.00	−0.01	0.02	0.04	−0.03	−0.03	0.07 ***	0.14 ***	-			
**Service utilization**															
13. Medicaid	0.13 ***	0.12 ***	0.09 ***	0.16 ***	−0.18 ***	−0.13 ***	0.45 ***	0.09 ***	0.21 ***	0.02	0.21 ***	0.05 *	-		
14. Outreach Programs	−0.00	−0.00	0.02	0.16 ***	−0.04	−0.03	0.09 ***	0.03	−0.02	−0.03	0.08 ***	−0.02	0.17 ***	-	
15. Dental Service	0.07	−0.01	0.01	0.00	−0.14 ***	−0.06	0.24 ***	−0.08 *	0.04	−0.05	−0.00	-	0.08	0.16 ***	-

* *p* < 0.05, ** *p* < 0.01, *** *p* < 0.001.

**Table 4:** Prevalence ratios (PRs) (95% CI) of the utilization of medical and dental services by three domains of Gelberg–Andersen behavioral model in homeless people, Seoul. Korea.

Variables	Unadjusted	Model 1	Model 2	Model 3
*Medicaid*				
** Predisposing**				
Sex (Men = 1)				
Women	1.22 (1.15−1.29) ***	1.10 (1.04−1.17) **	1.01 (0.96−1.07)	1.02 (0.96−1.08)
Age (< 50 = 1)				
≥50	1.19 (1.11−1.28) ***	1.16 (1.08−1.24) ***	1.06 (0.99−1.14)	1.05 (0.98−1.13)
Education (≥High School =1)				
≤Middle School	1.12 (1.06−1.18) ***	1.05 (0.99−1.11)	1.05 (0.99−1.11)	1.05 (0.99−1.11)
Duration (<10 years = 1)				
≥10	1.21 (1.15−1.28) ***	1.15 (1.08−1.21) ***	1.08 (1.02−1.14) **	1.07 (1.01−1.13) *
Drinking				
Yes	0.79 (0.74−0.84) ***	0.87 (0.81−0.92) ***	1.00 (0.94−1.06)	1.00 (0.94−1.07)
Smoking				
Yes	0.85 (0.80−0.89) ***	0.93 (0.88−0.99) *	0.96 (0.91−1.02)	0.97 (0.91−1.02)
** Enabling**				
Housing (Street = 1)				
Small Rooming	2.62 (2.11−3.25) ***		2.28 (1.76−2.95) ***	2.19 (1.69−2.84) ***
Shelter	3.37 (2.76−4.12) ***		2.53 (2.03−3.15) ***	2.45 (1.96−3.06) ***
Employed (yes = 1)				
No	1.13 (1.06−1.20) ***		1.06 (1.00−1.12) *	1.06 (1.00−1.12) *
Income (≥500 = 1)				
<500	1.32 (1.22−1.42) ***		1.16 (1.05−1.29) **	1.16 (1.05−1.29) **
** Need**				
Subjective Health				
Bad	1.03 (0.97−1.09)			1.02 (0.96−1.07)
Medical Diseases				
Yes	1.42 (1.30−1.55) ***			1.12 (1.03−1.21) **
Dental Diseases				
Yes	1.08 (1.01−1.14) *			1.00 (0.94−1.06)
Pseudo R^2^		0.0097	0.0308	0.0315
Outreach Programs				
** Predisposing**				
Sex (Men = 1)				
Women	1.00 (0.93−1.07)	0.92 (0.85−1.00)	0.91 (0.82−0.99) *	0.90 (0.82−0.99) *
Age (<50 = 1)				
≥50	0.99 (0.93−1.06)	0.97 (0.91−1.03)	0.96 (0.90−1.03)	0.97 (0.90−1.04)
Education (≥High school =1)				
≤Middle School	1.03 (0.97−1.09)	1.00 (0.94−1.06)	1.02 (0.96−1.09)	1.02 (0.96−1.09)
Duration (<10years = 1)				
≥10	1.22 (1.16−1.30) ***	1.23 (1.16−1.30) ***	1.23 (1.15−1.31) ***	1.22 (1.14−1.31) ***
Drinking				
Yes	0.95 (0.89−1.00)	0.97 (0.91−1.03)	0.99 (0.93−1.06)	0.99 (0.93−1.06)
Smoking				
Yes	0.97 (0.91−1.02)	0.99 (0.93−1.05)	0.95 (0.88−1.02)	0.95 (0.88−1.01)
** Enabling**				
Housing (Street = 1)				
Small Rooming	1.05 (0.93−1.18)		0.91 (0.78−1.05)	0.91 (0.78−1.05)
Shelter	1.17 (1.06−1.28) **		1.03 (0.93−1.14)	1.01 (0.92−1.12)
Employed (Yes = 1)				
No	1.05 (0.99−1.11)		1.04 (0.97−1.11)	1.04 (0.97−1.11)
Income (≥500 = 1)				
<500	0.98 (0.92−1.04)		0.88 (0.81−0.96) **	0.88 (0.81−0.96) **
** Need**				
Subjective Health				
Bad	0.96 (0.91−1.02)			0.96 (0.90−1.02)
Medical Diseases				
Yes	1.14 (1.06−1.22) **			1.05 (0.96−1.14)
Dental diseases				
Yes	0.98 (0.92−1.04)			0.96 (0.89−1.03)
Pseudo R^2^		0.0040	0.0048	0.0051
Dental service				
** Predisposing**				
Sex (Men = 1)				
Women	1.14 (0.98−1.32)	1.01 (0.85−1.21)	0.96 (0.79−1.17)	0.95 (0.78−1.17)
Age (<50 = 1)				
≥50	0.99 (0.84−1.16)	0.96 (0.82−1.13)	0.99 (0.84−1.18)	1.00 (0.84−1.18)
Education (≥High School = 1)				
≤Middle School	1.02 (0.89−1.16)	1.02 (0.88−1.17)	0.96 (0.83−1.12)	0.96 (0.83−1.12)
Duration (<10years = 1)				
≥10	1.01 (0.88−1.15)	0.99 (0.87−1.13)	0.96 (0.82−1.12)	0.97 (0.83−1.13)
Drinking				
Yes	0.79 (0.69−0.92) **	0.80 (0.69−0.94) **	0.83 (0.70−0.98) *	0.83 (0.70−0.98) *
Smoking				
Yes	0.91 (0.80−1.04)	0.93 (0.80−1.09)	0.91 (0.77−1.08)	0.90 (0.76−1.07)
** Enabling**				
Housing (Street = 1)				
Small Rooming	1.42 (0.96−2.08)		1.16 (0.70−1.90)	1.18 (0.72−1.95)
Shelter	1.93 (1.39−2.68) ***		1.54 (1.07−2.24) *	1.55 (1.07−2.25) *
Employed (Yes = 1)				
No	0.87 (0.77−0.99) *		0.82 (0.70−0.97) *	0.83 (0.71−0.98) *
Income (≥500 = 1)				
<500	1.08 (0.92−1.26)		1.00 (0.83−1.20)	0.99 (0.82−1.19)
** Need**				
Subjective Health				
Bad	0.91 (0.80−1.05)			0.94 (0.81−1.09)
Medical Disease				
Yes	1.00 (0.82−1.20)			0.93 (0.75−1.15)
Pseudo R^2^		0.0050	0.0144	0.0151

* *p* < 0.05, ** *p* < 0.01, *** *p* < 0.001.
